# Primary mature adrenal teratoma in infant

**DOI:** 10.11604/pamj.2020.37.27.24016

**Published:** 2020-09-07

**Authors:** Siham El Haddad, Laila Hessissen, Maria El Kababri, Najat Lamalmi, Mounir Kisra, Nazik Allali, Latifa Chat

**Affiliations:** 1 Pediatric Imaging Department, Pediatric Teaching Hospital, Rabat, Morocco,; 2 Pediatric Oncology Department, Pediatric Teaching Hospital, Rabat, Morocco,; 3 Pediatric Anatomical Pathology Department, Pediatric Teaching Hospital, Rabat, Morocco,; 4 Pediatric Surgery Department, Pediatric Teaching Hospital, Rabat, Morocco

**Keywords:** Primary mature, retroperitoneum, teratoma

## Abstract

Teratomas are neoplasms of the embryonic tissues that typically arise in the gonadal and sacrococcygeal regions. Primary adrenal teratoma are extremely rare and only few cases were published in literature. Teratomas contain more than one embryonic germ cell layer, mostly elements derived from ectoderm and least frequently from endoderm. Though these tumors are mostly benign, malignant transformation may occur. Treatment includes surgical removal. We report a rare case of a primary mature retroperitoneal teratoma in an infant with liver metastasis. Imaging modality CT and MRI were useful in diagnosis. The diagnostic and therapeutic challenges of dealing with such a case have been discussed and the literature reviewed.

## Introduction

Retroperitoneal teratomas (RPT) are the third most common retroperitoneal tumor in the pediatric population after neuroblastoma and Wilms tumor. Majority of the lesions are benign. Primary retroperitoneal teratoma are very rare in infant and malignancy with adrenal location is uncommon. These tumors represent a real challenge to the clinician, the radiologist and the surgeon. Most of the cases are incidentally detected or present an enlargement of the abdomen. Occasionally, the tumor is present antenatally and diagnosed at birth. Imaging based on ultrasound, computed tomography and resonance magnetic imaging remains important in the diagnostic and the extent of the lesion. The treatment is surgical excision. Prognosis is generally good following complete surgical. Chemotherapy may be helpful in some cases [1,2]. The present report is a case of a 12-month-old girl with an unusual teratoma in this age group and at this site. This case was a real challenge for the doctors.

## Patient and observation

**Figure 1:**
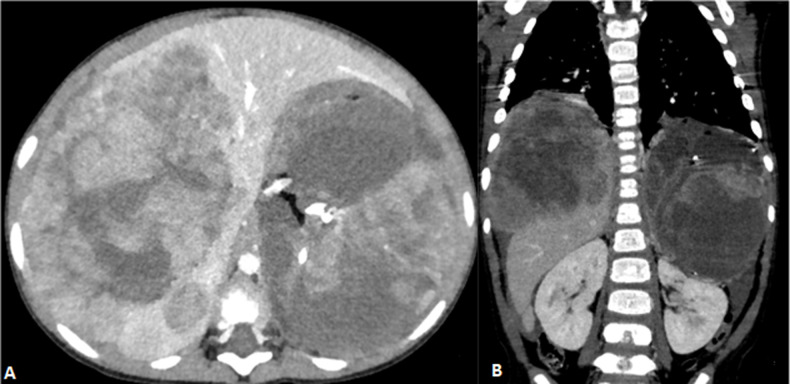
axial (A) and coronal CT (B) imaging showing a large left heterogeneous retroperitoneal mass with foci of calcification and fat. A large similar mass involving the VIII liver segment. Left kidney is displaced inferiorly and compressed against the posterior abdominal wall

**Figure 2:**
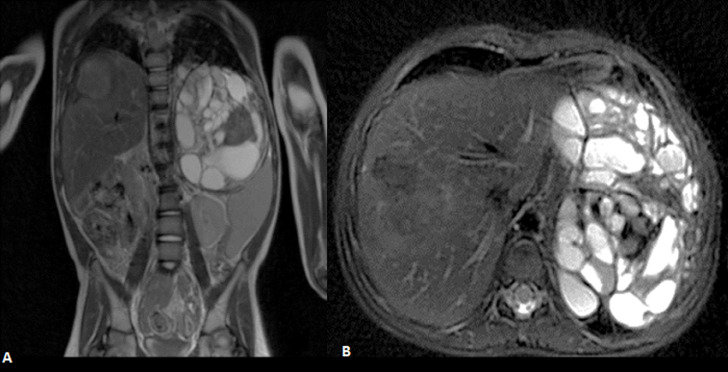
coronal (A) and axial (B) T2 weighted MR imaging showing a large left heterogeneous retroperitoneal mass with foci of calcification and fat. A large similar mass involving the VIII liver segment

**Figure 3:**
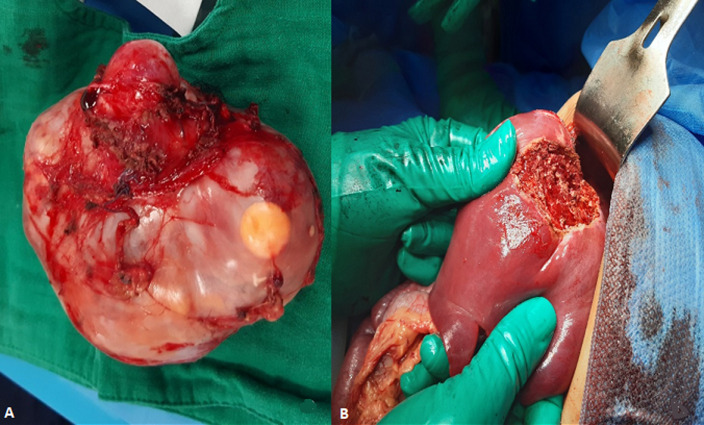
resected tumor image showed a large complex cystic and solid adrenal mass (A). Postoperative gross specimen showing the liver metastasis (B)

**Figure 4:**
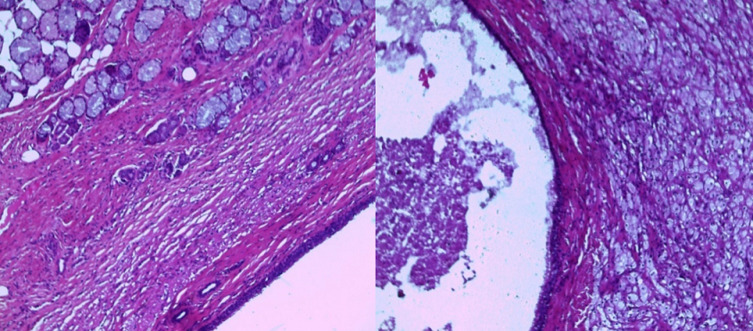
selected histology images demonstrate teratoma with post chemotherapy necrosis

A 01-year-old female born at term, product of normal pregnancy, was admitted with a progressively increasing mass in the abdomen. There was no notable family or past medical history. Her abdominal examination revealed a single non mobile, non-compressible mass involving left hypochondrium region. Hematological investigations found: serum alpha fetoprotein (AFP) was 40000 ng/ml and beta HCG (HCG) < 1,2 MUI/ML. Contrast enhanced computed tomography (CT) of abdomen revealed a large left well-defined heterogeneously enhancing mass lesion of size 120x 109 x85 mm. The lesion showed scattered areas of calcific and fat attenuation, vascularity in retroperitoneum and displacing surrounding viscera. A liver mass with the same characteristics was involving the VII, V, IV and VIII segments and measuring 125 x90 x 100 mm (Figure 1). No other lesion was found. The patient received six courses of chemotherapy (TGM 95 protocol) and the AFP evolution is detailed in Table 1. Control MR imaging revealed a tumor progression with a decrease of the liver lesion (Figure 2). Resection of tumor was done successfully. Intraoperative finding revealed a large retro- peritoneal tumor consisting of solid and cystic area occupying the left and central abdomen. The liver mass was also removed (Figure 3). The anatomopathological finding reveals a primary teratoma and the liver lesion was a metastasis (Figure 4). The patient has been doing well on 6 months follow-up with no clinical, radiological and biochemical evidence of any recurrence.

**Table 1:** table showing the evolution of the alpha-foeto-protein level during the chemotherapy process

AFP (ng/ml) Admission	After 2 cures VIP	After 4 VIP cures	After 6 cures of VIP and before surgery	After surgery	Actually
40000	8170	2180	441	8,89	3,47

## Discussion

Teratomas belong to the non seminomatous group of germ cell tumors, and are the commonest congenital neoplasms [3,4]. Mature teratoma usually contains two or three germ cell layers from the endoderm, mesoderm and ectoderm. Based on their composition, teratomas can be classified into solid, cystic, or mixed teratomas [4]. Extragonadal teratomas are is exceedingly rare tumors in neonates and infants and can sometimes show unusual, distinctive feature [1,5,6]. The involvement of extra-gonadal sites in decreasing order of frequency is mediastinum, sacrococcygeal region, retro peritoneum, pineal gland [3,7,8]. Retroperitoneal teratoma constitute 1-11% of primary retroperitoneal tumors [6,7,9]. Their incidence is bimodal with peaks in the first 6 months of life and in early adulthood [1]. Majority of the tumors are benign, situated on the left side and para renal in location. Occasional lesions are bilateral [10] but malignancy may be encountered [11]. Primary mature teratomas are uncommon and are made up of well-differentiated parenchymal tissues composed of somatic cell types that are derived from two or more germ layers [7,12]. The presence of nephroblastic components is extremely rare in retroperitoneal teratomas [1,9,12].

Teratomas are often asymptomatic as retro-peritoneal space is extensive enough to allow for their free growth [5]. Progressive enlargement of the abdomen and the presence of an intra-abdominal mass is the most common clinical feature [1,7]. Some teratomas can hide more or less extensive islands of immaturity signs of malignant transformation that are clinically evident [5]. They are believed to arise as metastasis from the gonadal tissue rather than to represent true primary tumors. Only a very few case reports have been documented in literature so far [12] which make our case unusual. An accurate diagnosis of a teratoma cannot be made on clinical basis [2,7]. Diagnosis can be possible with the help of plain radiography, ultrasound abdomen and computed tomography (CT) or magnetic resonance image (MRI) of abdomen [8]. Radiological features include presence of calcification, teeth and fat; however, calcification cannot be considered an indicator of a benign tumor. Ultrasound of the abdomen is usually the first imaging modality used in pediatric [7,8]. It can greatly differentiate between cystic and solid elements [2,12]. CT scan and MRI are useful to delineate the extent of the disease in retro-peritoneum and its relationship to major vessels, which provide better preoperative planning and increased likelihood of complete removal of the tumor with less iatrogenic damage [10]. Internal homogeneous, fat density, cyst formation and calcification are considered important predictor of a benign retro peritoneal teratoma on CT scan [7]. MRI scans can show better resolution of soft tissues, feasible identification of benign and malignant neoplastic features, and most importantly superior tumor staging assessment 1 [2].

Retroperitoneal teratomas can express a diversity of serum tumor markers such as elevated AFP, carcinoembryonic antigen (CEA), and CA 19-9. Serum alpha-feto protein level is good indicator for diagnosis and assessing the recurrence of tumor [7,11,13]. The differential diagnosis of retroperitoneal teratoma in infant includes nephroblastoma, neuroblastoma, ovarian tumors and lymphangioma [14]. The prognosis is generally good and curative if the tumor is completely removed [5,3,10]. However, the prognosis of some patients with teratoma malignant transformation was poor [14]. Complete surgical excision provides the best chance of cure and is the most important prognostic factor [7,11,14]. The treatment may associate, if necessary, with adjuvant chemotherapy [5,11,13]. Finally, to improve the prognosis, close, long-term clinical, laboratory and imaging surveillance is necessary, at shorter intervals during the first 5 years after the exeresis and annually thereafter [5]. Our patient received chemotherapy then surgery and has been doing well by 06 months.

## Conclusion

Adrenal teratomas are extremely rare and primary adrenal teratomas are even rarer. Cross sectional imaging based on ultrasonography, CT and MRI is very helpful in the pre-operative diagnosis and distinguishing them from similar characteristic retroperitoneal lesions.

Retroperitoneal laparoscopic surgery is the preferred treatment for adrenal teratoma.
